# The effects of passive leg raising may be detected by the plethysmographic oxygen saturation signal in critically ill patients

**DOI:** 10.1186/s13054-019-2306-z

**Published:** 2019-01-18

**Authors:** Alexandra Beurton, Jean-Louis Teboul, Francesco Gavelli, Filipe Andre Gonzalez, Valentina Girotto, Laura Galarza, Nadia Anguel, Christian Richard, Xavier Monnet

**Affiliations:** 1 Service de réanimation-médecine intensive, Centre Hospitalier Universitaire de Bicêtre, Hôpitaux universitaires Paris-Sud, Assistance publique – Hôpitaux de Paris, 78, rue du Général Leclerc, F-94 270 Le Kremlin-Bicêtre, France; 2 Inserm UMR S_999, Université Paris-Sud, Le Kremlin-Bicêtre, France

**Keywords:** Fluid responsiveness, Volume expansion, Cardiac index, Perfusion index, Oxygen saturation

## Abstract

**Background:**

A passive leg raising (PLR) test is positive if the cardiac index (CI) increased by > 10%, but it requires a direct measurement of CI. On the oxygen saturation plethysmographic signal, the perfusion index (PI) is the ratio between the pulsatile and the non-pulsatile portions. We hypothesised that the changes in PI could predict a positive PLR test and thus preload responsiveness in a totally non-invasive way.

**Methods:**

In patients with acute circulatory failure, we measured PI (Radical-7) and CI (PiCCO2) before and during a PLR test and, if decided, before and after volume expansion (500-mL saline).

**Results:**

Three patients were excluded because the plethysmography signal was absent and 3 other ones because it was unstable. Eventually, 72 patients were analysed. In 34 patients with a positive PLR test (increase in CI ≥ 10%), CI and PI increased during PLR by 21 ± 10% and 54 ± 53%, respectively. In the 38 patients with a negative PLR test, PI did not significantly change during PLR. In 26 patients in whom volume expansion was performed, CI and PI increased by 28 ± 14% and 53 ± 63%, respectively. The correlation between the PI and CI changes for all interventions was significant (*r* = 0.64, *p* < 0.001). During the PLR test, if PI increased by > 9%, a positive response of CI (≥ 10%) was diagnosed with a sensitivity of 91 (76–98%) and a specificity of 79 (63–90%) (area under the receiver operating characteristics curve 0.89 (0.80–0.95), *p* < 0.0001).

**Conclusion:**

An increase in PI during PLR by 9% accurately detects a positive response of the PLR test.

**Trial registration:**

ID RCB 2016-A00959-42. Registered 27 June 2016.

**Electronic supplementary material:**

The online version of this article (10.1186/s13054-019-2306-z) contains supplementary material, which is available to authorized users.

## Background

Volume expansion is often the first-line treatment used to increase cardiac index (CI) in patients with acute circulatory failure [1]. However, increasing cardiac preload with fluid administration does not always induce the increase in CI that was expected from it. Moreover, excessive fluid loading with positive cumulative fluid balance may have deleterious effects and impair prognosis of critically ill patients, especially in cases of septic shock [2, 3] and acute respiratory distress syndrome [4, 5].

If preload responsiveness is not obvious, as in the case of fluid loss or at the initial phase of septic shock, it is crucial to predict the response of cardiac output to fluid administration before performing it. The passive leg raising (PLR) test is one of the methods currently available for this purpose [1]. It consists of moving the patient from the semi-recumbent position to a position in which the trunk is horizontal and the inferior limbs are passively elevated at 45° [6]. The PLR test induces the transfer of some venous blood from the lower part of the body toward the cardiac cavities. It increases the mean systemic pressure [7], resulting in an increase in the pressure gradient of venous return and in CI in preload responsive patients [1, 8]. The test has been demonstrated to be reliable by many studies and two meta-analyses [9, 10]. Nevertheless, to detect the changes in CI induced by a PLR test, a direct and real-time measurement of CI is needed [6], which is often invasive.

The peripheral perfusion index (PI) is derived from the plethysmographic signal of pulse oximetry, which is obtained from the amount of infrared (940 nm) light transmitted through the vascular bed of a finger. The plethysmographic signal has two components. The pulsatile component reflects changes in the finger blood volume during one cardiac cycle, which may depend on the changes in stroke volume [11], while the non-pulsatile component is related to the light absorbed by the other tissues, such as connective tissue, bone, venous and capillary blood [12, 13]. Some plethysmographic devices like the Radical-7 (Masimo Corp., Irvine, CA, USA) automatically calculate the ratio of the pulsatile over the non-pulsatile component of the plethysmographic signal, which is called PI and reflects the quality of skin perfusion [12–15]. Then, the changes in the ratio of pulsatile over non-pulsatile component of the plethysmographic signal may depend on the changes in CI.

In this context, monitoring PI might be an attractive method for assessing the effects of the PLR test when no direct measurement of CI is available. The goal of our study, conducted in critically ill patients with acute circulatory failure, was to test if PI changes could accurately detect a positive response of CI to a PLR test.

## Methods

### Patients

This prospective study was conducted in the 25-bed medical intensive care unit of a university hospital. It was approved by the Institutional Review Board of our institution (Comité pour la Protection des Personnes, Ile-de-France VII, ID RCB: 2016-A00959-42). All patients or their relatives accepted to participate in the study. They were included if they were older than 18 years old, if they were routinely equipped with a PiCCO2 device (Pulsion Medical Systems, Feldkirchen, Germany) and if clinicians in charge decided to perform a PLR test. Fifty (69%) patients were included during the resuscitation or stabilisation phase of shock, defined by an increase in the dose of norepinephrine during the last 24 h, and 22 (31%) patients were included at the de-resuscitation phase, defined by a decrease in the dose of norepinephrine over the last 24 h. Patients were excluded *a priori* if the PLR test was contraindicated (head trauma, deep vein thrombosis in the inferior limbs) or supposed to be unreliable (venous compression stocking, intra-abdominal hypertension, defined as an intra-abdominal pressure (IAP) > 12 mmHg [16]) and *a posteriori* if the plethysmographic signal was absent and unstable. Plethysmographic signal instability was defined by a precision of PI ≥ 10%.

### PiCCO2 device and haemodynamic variables

The PiCCO2 system is composed of a central jugular venous catheter and a thermistor-tipped arterial femoral catheter (PC8500, Pulsion Medical Systems, Feldkirchen, Germany) that are connected to a specific device. CI was measured by calibrated pulse contour analysis [17] and by transpulmonary thermodilution [18]. Transpulmonary thermodilution measurements were performed by the injection of cold boluses of 15 mL of 0.9% saline into the central venous tract. The average of three consecutive measurements was recorded and averaged [19]. The systemic arterial and central venous pressure curves were recorded continuously by using a data acquisition software (HEM 4.2, Notocord, Croissy-sur-Seine, France). CI was continuously recorded by the PiCCO Win 4.0 software (Pulsion Medical Systems). We measured IAP from the bladder pressure by injecting 25 mL of saline in the bladder after clamping the urinary drainage bag (AA6118 FOLYSIL, Humlebaek, Denmark). The abdominal pressure transducer was fixed to the patient on the lateral side of the pelvis, 2 cm below the anterior superior iliac spine. IAP was measured at end-expiration, in the absence of abdominal muscle contractions, which was checked by clinical examination. We defined intra-abdominal hypertension as IAP ≥ 12 mmHg [16].

### Perfusion index

PI was automatically calculated from the plethysmogram by the Radical-7 device as the ratio between the amplitude of the pulsatile and the non-pulsatile components of the light received by the detector of the pulse oximeter, expressed as a percentage. It was measured by a sensor placed on the third or fourth finger, by choosing the one with the highest PI value, as recommended by the constructor. If no signal was obtained on these fingers, we did not attempt to obtain a signal at another site of measurement. The device offers two methods for displaying PI values. With the “short-time” method, PI is displayed in real time with no averaging. With the “long-time” method, the displayed PI values result from a 30-s moving average. We chose the “short-time method” and averaged the PI values over 12 s because it is the same time that is used by the PiCCO2 device for averaging pulse contour analysis-derived CI values.

### Study design and measurements

Immediately after inclusion of the patients, when patients were in the semi-recumbent position, we collected demographic characteristics, PI and haemodynamic variables, including heart rate and arterial and central venous pressure. Stroke volume index (SVI) and CI were measured by transpulmonary thermodilution. The pressure sensors connected to the arterial and central venous catheters were fixed on the upper arm of the patient at the estimated level of the right atrium. A PLR test was then performed by transferring the patient to the PLR position, in which the lower limbs are passively elevated at 45° and the trunk is horizontal [6]. When the PLR test had induced its maximal effect on CI, which occurs within 1 min, we performed another set of measurements including CI. At this time, CI was measured by pulse contour analysis, because the effects of PLR may decrease after reaching their maximum in some patients, so that transpulmonary thermodilution may miss the maximal effects because of the time required for performing three boluses injections [6]. Then, we moved the patient back to the semi-recumbent position. We performed a third set of measurements, including heart rate, arterial and central venous pressure, PI, SVI and CI measured by transpulmonary thermodilution.

The PLR test could be planned in view of infusing fluid. In such cases, in case of a positive PLR test, clinicians could decide to perform a volume expansion with 500 mL of saline, weighting its risks and benefits. The PLR test could also be performed for guiding the decision of fluid removal [20]. In such cases, a negative PLR test could lead to fluid removal, again depending on the decision of the clinicians in charge. Immediately after the end of fluid infusion, we performed the last set of measurements of mean arterial pressure, heart rate, central venous pressure, PI, SVI and CI (transpulmonary thermodilution). Catecholamines and sedative drugs doses as well as ventilation settings were kept constant during the study.

### Measurement of the precision of PI

In each patient, during a period of time when the haemodynamic status was stable (change in pulse contour analysis-derived CI < 10%), we recorded five successive values of PI, each averaged over 12 s. During this time, the position of the plethysmographic sensor was kept unchanged. We calculated the coefficient of variation of PI as being the standard deviation divided by the mean of the five measurements [17–19].

The coefficient of variation is a relative measure of the dispersion of the data around the mean. It allows the comparison of the degree of variation from one sample to another, even if the averages are different. The precision was calculated as being two times the coefficient of variation and the least significant change as the coefficient of variation × 1.96 × √2 [19–21]. The least significant change is the most interesting variable to observe since it indicates the minimum change measured by the device that can be trusted as a real change of measurement [22]. It must be compared to the changes that have been actually observed during the study.

### Statistical analysis

The PLR test was defined as positive if it increased CI ≥ 10%. The response to volume expansion was defined as positive if CI increased ≥ 15% just after fluid administration. Data were expressed as mean ± standard deviation, median [interquartile range, IQR] and number (percentage). Normality was assessed by the Kolmogorov-Smirnov test. Pairwise comparisons of values between different study times were performed by paired Student *t* tests. Comparisons between patients with positive PLR and patients with negative PLR tests were performed by two-tailed Student *t* tests or the Wilcoxon test.

We compared the relative changes of CI to those of PI by linear regression analysis (for percent changes). To assess the trending ability of PI, we constructed a regression curve. This allowed the calculation of the percentage of total data points for which the directional changes of PI were concordant with those of CI. Correlations were assessed by the Spearman coefficient. Receiver operating characteristic (ROC) curves (with 95% confidence interval) were generated for describing the ability of the PLR-induced percent changes in PI to detect the PLR-induced percent changes in CI. The areas under ROC curves were compared by the Hanley-McNeil test [23]. The Youden index was calculated as sensitivity + specificity − 1 and was used to determine the diagnostic threshold.

The calculation of the sample size was based on the areas under the ROC curves. Considering a null hypothesis at 0.75, expecting an area under the curve for the PLR-induced changes in PI of 0.90 and taking into account an *α* risk at 5% and a *β* risk at 20%, we planned to include 34 patients per group. Statistical analysis was performed using MedCalc 11.6.0 software (Mariakerke, Belgium).

## Results

### Patient characteristics

We initially screened 85 patients which the characteristics are detailed in Table 1. Among the 85 screened patients, 7 were excluded because of intra-abdominal hypertension (IAP, 18 ± 3 mmHg). Three other ones were excluded because the plethysmographic signal was not obtained. Their characteristics were not different from the other ones in terms of arterial pressure, dose of norepinephrine or shock origin (septic for 8 patients and hypovolemic for 2). Three patients presented an unstable plethysmography signal. Two of these 3 patients were the only screened ones who presented atrial fibrillation. No patient presented frequent atrial or ventricular extrasystoles. Eventually, 72 patients were included. A flow chart is displayed in Additional file 1: Figure S1.

**Table 1:** Patient characteristics (*n* = 72)

Age (mean ± SD, years)	64 ± 13
Gender (male, *n* (%))	56 (77%)
Weight (mean ± SD, kg)	72 ± 16
Height (mean ± SD, cm)	168 ± 10
SAPS II (mean ± SD)	60 ± 20
Type of shock (*n* (%)):	
Septic	51 (70%)
Cardiogenic	12 (17%)
Hypovolemic	9 (13%)
Catecholamines	
Norepinephrine (*n* (%))	52 (72%)
Dose of norepinephrine (median [interquartile range], μg/kg/min)	0.5 [0.1–0.6]
Dobutamine (*n* (%))	8 (11%)
Dose of dobutamine (median [interquartile range], μg/kg/min)	16 [14–20]
Respiratory settings	
Mechanical ventilation (*n* (%))	56 (78%)
Tidal volume (mean ± SD, mL/kg of PBW)	5.8 ± 1.4
Plateau pressure (mean ± SD, cmH_2_O)	23.5 ± 3.8
Positive end-expiratory pressure (mean ± SD, cmH_2_O)	9.8 ± 3.5

Values are expressed as mean ± standard deviation, number (*n*) and frequency (%) or median and interquartile range

*PBW* predicted body weight, *SAPS II* Simplified Acute Physiology Score, *SD* standard deviation

No patient had *acute cor pulmonale* or severe valvular disease. The IAP was 4 ± 3 mmHg. Among the 20 (28%) patients who had no norepinephrine at the time of inclusion, it had been stopped in 13 (18%) patients, who were in the stabilisation phase of their disease, and it had never been administered before in 7 (10%) patients. The lactate level at the time of inclusion was 1.8 ± 1.1 mmol/L. Most of the patients (56 (78%)) were mechanically ventilated.

### PI absolute values

At baseline, the value of PI was 2.5 ± 1.9%, ranging from 0.2 to 6.7%. It was lower than 1% in 23 patients. The value of PI at baseline was correlated with the dose of norepinephrine (*r* = − 0.29, *p* = 0.04). The value of PI was similar in patients with and without norepinephrine infusion (2.5 ± 1.9% vs. 2.2 ± 1.8%, respectively, *p* = 0.39), as well as in patients with and without mechanical ventilation (2.5 ± 1.9% vs. 1.6 ± 1.7%, respectively, *p* = 0.56). It was also similar between patients receiving the lowest and the highest doses of norepinephrine, as defined according to its median value (2.6 ± 2.0% vs. 2.6 ± 2.0%, respectively, *p* = 0.56) (Table 1). The absolute value of PI at different study times was correlated with mean (*r* = 0.20, *p* = 0.003) and with diastolic arterial pressure (*r* = 0.16, *p* = 0.01).

In the subgroup of patients with PI ≤ 1%, the dose of norepinephrine was similar as in the other ones (0.5 ± 0.7 vs. 0.4 ± 0.3 μg/kg/min, respectively, *p* = 0.72). These patients with PI < 1% did also not differ in terms of lactate level at baseline (1.9 ± 1.1 vs. 1.7 ± 1.1 mmol/L, respectively, *p* = 0.49), time elapsed between the onset of shock and the inclusion (120 ± 108 vs. 98 ± 53 h, respectively, *p* = 0.44) or Simplified Acute Physiology Score (SAPS) II score (62 ± 21 vs. 56 ± 18, respectively, *p* = 0.43).

### Effects of PLR and volume expansion on PI

**Fig. 1:**
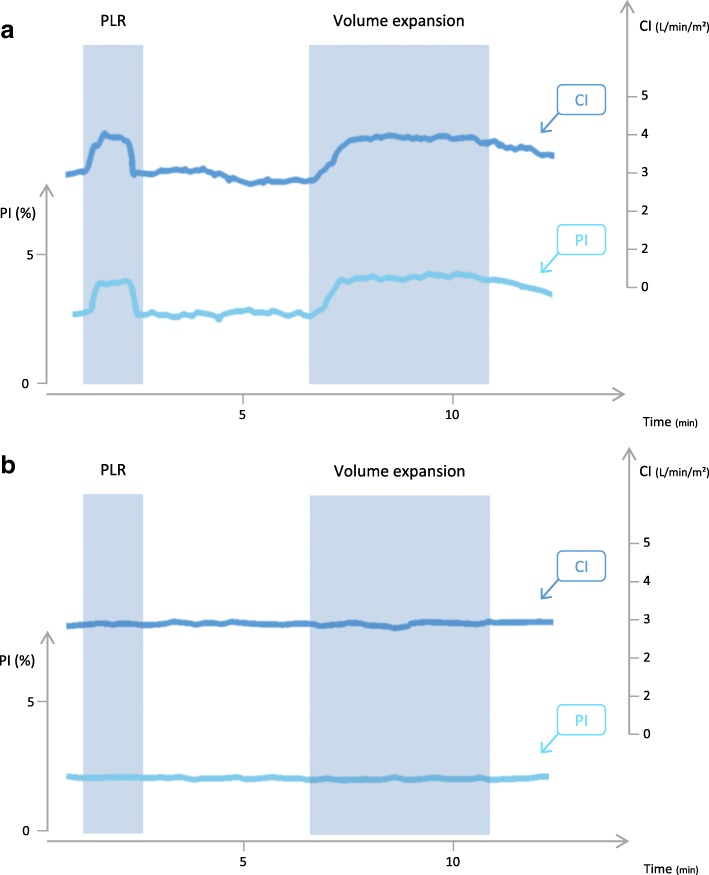
Typical waveform of perfusion index (PI), cardiac index (CI) signals during a passive leg raising (PLR) test and a volume expansion (VE) in preload responders (**a**) and in preload non-responders (**b**)

**Fig. 2:**
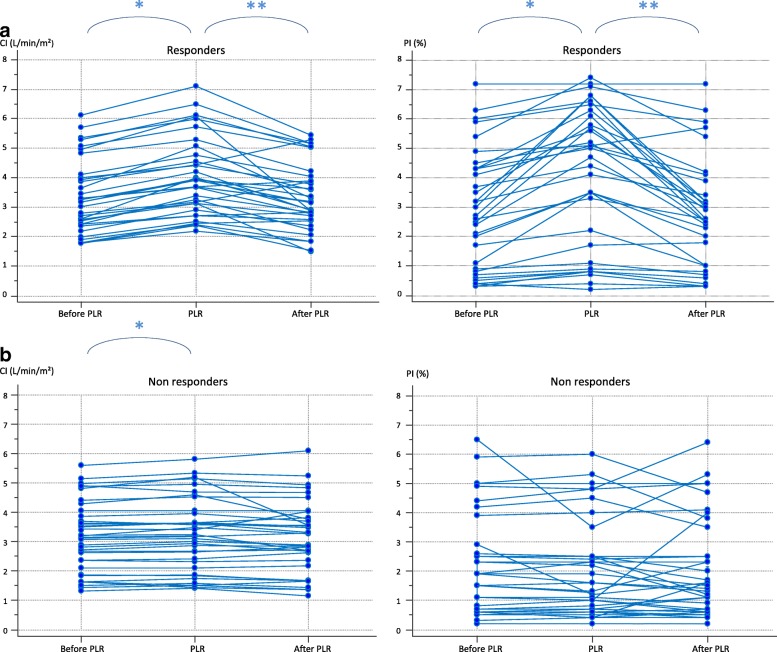
Changes in perfusion index (PI) and cardiac index (CI) during a passive leg raising (PLR) test in responders (*n* = 34) (**a**) and in non-responders (*n* = 38) (**b**)

The haemodynamic variables and their time course are reported in Table 2. The changes in CI and PI during a PLR test in a typical fluid responder and a typical non-responder are displayed in Fig. 1. The PLR test was positive (PLR-induced increase in CI ≥ 10%) in 34 patients. The PI value at baseline was similar in these patients and in the other ones (Table 2). In patients in whom the PLR test was positive, CI, SVI and PI significantly increased during PLR by 21 ± 10%, 18 ± 19% and 54 ± 53%, respectively (*p* < 0.001 for both) (Fig. 2). The PLR test was negative (CI increased by < 10%) in 38 patients. In these patients, CI and SVI significantly increased during PLR by 2 ± 4% and 2 ± 7%, respectively, and PI did not significantly change.

**Table 2:** Haemodynamic variables

	Baseline 1	PLR test	Baseline 2	After volume expansion
Heart rate (beats/min)				
• Positive PLR test (*n* = 34)	94 ± 16	95 ± 20	97 ± 17	93 ± 15^†^
• Negative PLR test (*n* = 38)	90 ± 20	89 ± 20	88 ± 20	
Systolic arterial pressure (mmHg)				
• Positive PLR test (*n* = 34)	115 ± 23	128 ± 29*	112 ± 28	122 ± 34^†^
• Negative PLR test (*n* = 38)	125 ± 20	130 ± 21*	123 ± 20	
Diastolic arterial pressure (mmHg)				
• Positive PLR test (*n* = 34)	58 ± 11	63 ± 10*	57 ± 13	61 ± 15^†^
• Negative PLR test (*n* = 38)	60 ± 9	63 ± 9*	59 ± 9	
Mean arterial pressure (mmHg)				
• Positive PLR test (*n* = 34)	77 ± 13	87 ± 21*	75 ± 17	80 ± 19^†^
• Negative PLR test (*n* = 38)	81 ± 12	85 ± 13*	80 ± 12	
Central venous pressure (mmHg)				
• Positive PLR test (*n* = 34)	10 ± 5	13 ± 5*	10 ± 4	11 ± 5^†^
• Negative PLR test (*n* = 38)	10 ± 5	14 ± 5*	9 ± 5	
Cardiac index (L/min/m^2^)				
• Positive PLR test (*n* = 34)	3.38 ± 1.21	4.03 ± 1.31*	3.20 ± 1.20	4.02 ± 1.35^†^
• Negative PLR test (*n* = 38)	3.19 ± 1.26	3.26 ± 1.32*¨	3.15 ± 1.31	
GEDV (mL/m^2^)				
• Positive PLR test (*n* = 34)	766 ± 165		768 ± 205	789 ± 127^†^
• Negative PLR test (*n* = 38)	800 ± 242		792 ± 219	
SVI (mL/m^2^)				
• Positive PLR test (*n* = 34)	37 ± 13	44 ± 15*	36 ± 12	43 ± 15^†^
• Negative PLR test (*n* = 38)	37 ± 15	38 ± 16	37 ± 17	
PPV (%)^‡^				
• Positive PLR test (*n* = 28)	10.0 ± 4.0		9.8 ± 5.9	11.9 ± 16.2
• Negative PLR test (n = 28)	8.0 ± 5.1		8.5 ± 4.7	
PI (%)				
• Positive PLR test (*n* = 34)	2.9 ± 2.0	4.1 ± 2.3*	2.1 ± 1.4	3.0 ± 1.9^†^
• Negative PLR test (*n* = 38)	2.0 ± 1.8	2.0 ± 2.0¨	2.1 ± 1.9	

Values are expressed as mean ± standard deviation

*PLR* passive leg raising test, *GEDV* global end-diastolic volume, *SVI* stroke volume index, *PPV* pulse pressure variation, *PI* perfusion index

*p < 0.05 vs baseline 1

^†^*p* < 0.05 vs baseline 2

¨*p* < 0.05 between positive and negative PLR test

^‡^In mechanically ventilated patients

Volume expansion was decided after 27 positive PLR tests. Twenty-six of these patients were eventually fluid responders (fluid-induced increase in CI ≥ 15%). In these patients, after volume expansion, CI and PI significantly increased by 28 ± 14% and 53 ± 63%, respectively (*p* < 0.001 for both). Only 1 patient with a positive PLR test was a fluid non-responder. In this patient, the fluid-induced increase in CI was 9%, whereas the PLR-induced increase in CI was 15%.

### Ability of PI changes to detect a positive PLR test

**Fig. 3:**
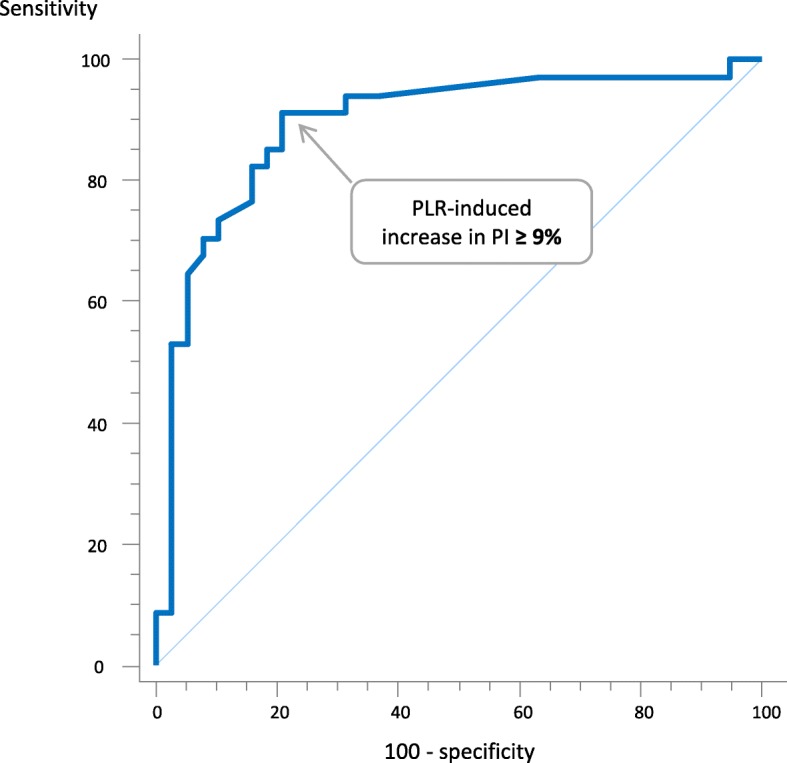
Area under the receiver operating characteristics curve generated for the detection of a positive passive leg raising (PLR) test by the changes in perfusion index (PI). The Youden index was calculated as sensitivity + specificity − 1

During PLR, if PI increased by > 9%, a positive response of CI (increase by > 10%) to PLR could be diagnosed with a sensitivity of 91% (76–98%), a specificity of 79% (63–90%), a positive predictive value of 80% (64–91%) and a negative predictive value of 91% (76–98%). The area under the ROC curve was 0.89 (0.80–0.95) (*p* < 0.0001 vs. 0.5) (Fig. 3). PI increased by > 9% in 31 patients under 34 with a positive PLR.

In the subgroup of patients with PI ≤ 1%, the area under the ROC curve was similar to the area under the ROC curve in patients with PI > 1% ((0.94 (0.75–0.99) vs. 0.88 (0.77–0.96), respectively, *p* = 0.48).

### Ability of PI changes to track changes in CI and SVI

**Fig. 4:**
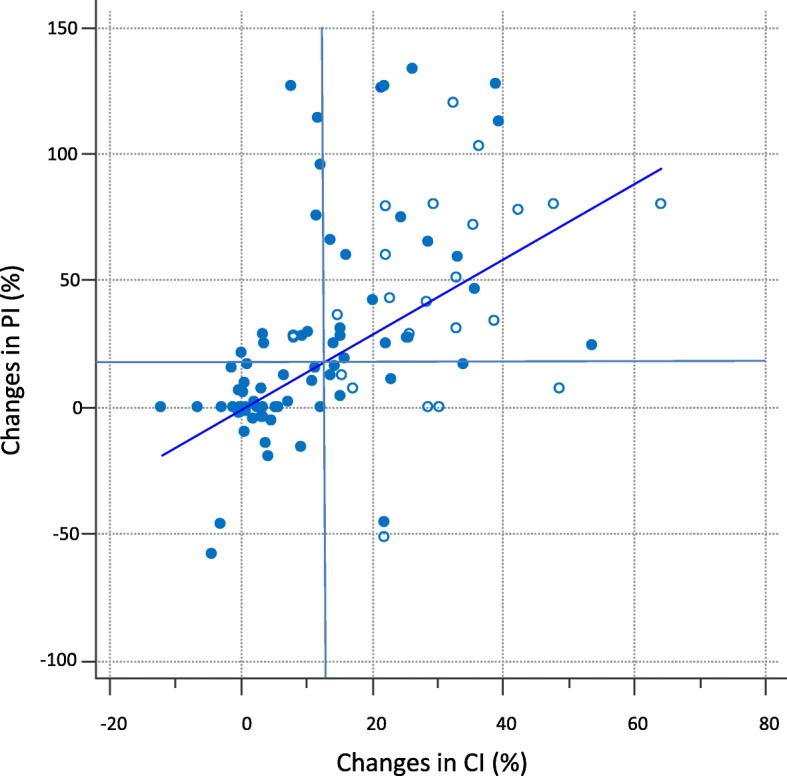
Correlation between perfusion index (PI) changes and cardiac index (CI) changes during passive leg raising () and volume expansion ()

The changes in PI and the changes in CI were correlated when considering all interventions (PLR in 72 patients and volume expansion in 27 patients) (*r* = 0.63, *p* < 0.0001, concordance rate = 73%, Fig. 4) or PLR only (*r* = 0.64, *p* < 0.0001). This was also the case for the changes in PI and the changes in SVI when considering all interventions (*r* = 0.26, *p* = 0.02, concordance rate = 59%) or PLR only (*r* = 0.33, *p* < 0.005, concordance rate = 63%).

### Precision of PI measurements

In included patients, the mean of PI values in measurements performed for assessing the precision was 1.37 ± 0.03% (in absolute value). In these patients, the coefficient of variation was 3.2%, the precision of PI was 1.2% and the LSC was 1.6%.

In patients excluded due to the instability of the PI signal, the mean of PI values in measurements performed for assessing the precision was 1.3 ± 0.2%. In these patients, the precision of PI was 14%, and the LSC was 19%.

## Discussion

This study suggests that, in patients in whom PI could be measured reliably, the increase in PI detected a positive response of CI to PLR with good accuracy.

The PLR test is an easy and reliable method to predict fluid responsiveness [9, 24] which is now accepted in clinical practice [1, 10, 25]. Nevertheless, its main drawback is that, like the fluid challenge [26, 27], its effects must be assessed by a direct measurement of CI, which must be precise and able to detect short-term changes with precision [6]. Our study suggests that the changes in PI might be used as a surrogate of the changes in CI during PLR and then could be used to assess preload responsiveness with an acceptable accuracy.

PI has been proposed to reflect the quality of skin perfusion, in particular in anaesthesia [13–15, 28]. Nonetheless, stroke volume should also influence the PI by increasing the arterial blood volume in the finger at each cardiac beat. The relationship between PI and CI cannot be straightforward because it is also influenced by the venous blood flow. A decrease in venous blood flow might cause a stagnation of venous blood in the fingers, an increase in the non-pulsatile component and eventually a decrease in PI independently from the changes in arterial blood flow.

Some studies have suggested that changes in PI reflect changes in CI [11, 29] or in the amplitude of arterial pressure [30] in various settings. Desgranges et al. have shown that the changes in PI measured at the forehead were able to detect the fluid-induced changes in CI with reliable accuracy [31] after induction of anaesthesia. Discrepant results have also been reported [28, 31, 32]. In particular, Broch et al. did not find any significant change in PI during a PLR manoeuvre [28]. Nonetheless, in all these negative studies, the quality of the PI was not mentioned.

Clearly, our results show that PI is not a perfect surrogate of CI. First, the plethysmographic signal was absent in three patients and was unstable in three other ones. Moreover, except in two patients with atrial fibrillation, there was no clear explanation for this instability. Second, the correlation between the changes in PI and changes in CI was not perfect. As stated above, this could be easily explained by the fact that other factors than CI influence the amplitude of the plethysmographic signal.

One may intuitively think that the low values of PI, the amplitude of which was very variable at baseline, are encountered in patients with the strongest vasoconstriction. Nevertheless, the PI value was not correlated with the dose of norepinephrine, and the PI value at baseline was not different between patients with the highest and the lowest doses of norepinephrine. Even in patients with PI values < 1%, the dose of norepinephrine was not different from that of the other ones. This does not exclude the fact that sepsis-related local hypoperfusion or local vasoconstriction was more pronounced in patients with low PI.

In spite of these limitations, we do think that our data provide new and interesting information. First, we describe a means of assessing the PLR effects that is easy to use, totally non-invasive and available in all patients without any additional cost. Second, in patients with a stable signal, PI was very precise. The precision was much lower than the threshold found to detect a positive PLR test, what means that the PI is a suitable surrogate of CI for this purpose. Third, PI might be used to reflect CI changes in other clinical circumstances than the PLR test, in which no cardiac output monitoring is available. Fourth, PI might be used even if its value is low (≤ 1%).

There are several limitations to our study. First, patients with a negative PLR test did not receive fluids, so that we could not conclude regarding the ability of PLR-induced changes in PI to predict fluid responsiveness. Nevertheless, the reliability of the PLR test should be considered as well established [9]. In the present study, only one patient in whom the PLR test was positive did not respond to fluid administration. In this patient, the PLR-induced increase in CI was close to 10%, and the fluid-induced increase in CI was also close from 15%. Second, we included only critically ill patients while the results regarding PI might differ in other contexts, especially because PI is influenced by skin perfusion, which might for instance be different in the perioperative period [30–32]. Third, we investigated only the PI at the finger level, while its relationship to stroke volume might differ among the site where it is measured [29, 31]. Fourth, we averaged the real-time value of PI over 12 s, which is not performed by the commercial version of the device. Nevertheless, we think this was the only way allowing a comparison with pulse contour analysis-derived CI, which is averaged over 12 s. Moreover, we did not test the “long average” version of the device, which averages PI over 30 s.

## Conclusions

In critically ill patients in whom it could be measured, the changes in PI during PLR test appear a reliable way to assess the haemodynamic effects of the PLR test, and thus to assess preload responsiveness, in a totally non-invasive way. This proof of concept opens the door for further investigations.

## Additional file

Additional file 1: **Figure S1.** Flowchart (*n* = 85). (PPTX 73 kb)

## Abbreviations

CI: Cardiac index
IAP: Intra-abdominal pressure
LSC: Least significant change
PLR: Passive leg raising
PPV: Pulse pressure variation
ROC: Receiver operating characteristics
SVI: Stroke volume index
