# Supplementary material for: The effects of passive leg raising may be detected by the plethysmographic oxygen saturation signal in critically ill patients
Source: Crit Care. 2019 Jan 18;23:19. doi: 10.1186/s13054-019-2306-z (PMC6339274; doi:10.1186/s13054-019-2306-z)
Supplement: Supplementary file 1 — Figure S1. Flowchart (n = 85). (PPTX 73 kb) [file 13054_2019_2306_MOESM1_ESM.pptx]

## Slide 1
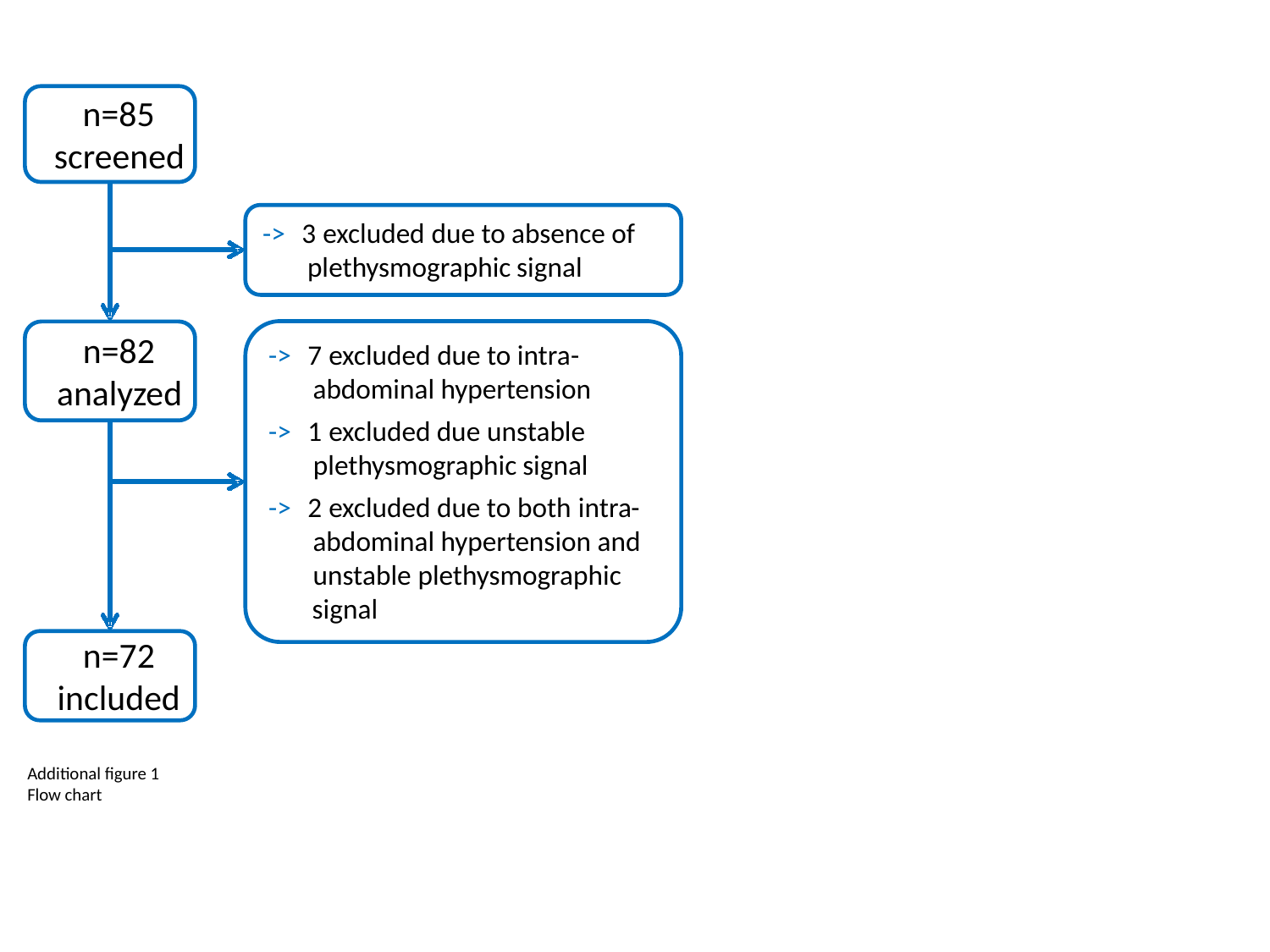

n=85
screened
->
3
excluded
due to absence of
plethysmographic
signal
n=82
->
7
excluded
due to intra
-
analyzed
abdominal hypertension
->
1
excluded
due
unstable
plethysmographic
signal
->
2
excluded
due to
both
intra
-
abdominal hypertension and
unstable
plethysmographic
signal
n=72
included
Additional figure 1
Flow chart
